# Phosphorus in Rickets

**Published:** 1890-06-14

**Authors:** 


### THE PRACTITIONER'S MIRROR AND RETROSPECT.

*[The Editor will be glad to receive offers of co-operation and contributions from members of the profession. There is no doubt that much valuable material full of interest and instruction is at present lost to science. This is largely so in the case of (1) the younger and less-known members of the hospital staff (2) those connected with cottage hospitals, and (3) the great body of medical practitioners not attached to any institution. The co-operation of all is cordially invited to enable the Editor to make* The Hospital *of the utmost possible use and value to the profession. Specialists willing to review books on their special subjects are invited to communicate this fact at once. All letters should be addressed to* The Editor, The Lodge, Porchester Square, London, W.]

### MEDICINE.

### PHOSPHORUS IN RICKETS.

The treatment of rickets has always been more or less empirical, consisting mainly in measures calculated to improve the general nutrition. Many drugs have been employed with more or less successful results, but none have had any claims to be considered as specific or as affecting directly the pathological conditions in which the disease consists.

Wegner's classical experiments pointed to the possession of such a character by phosphorus, but there has been much difference of opinion among physicians as to its clinical employment and therapeutic value. Kassowitz repeating the experiments of Wegner observed that when given in relatively large doses it caused a notable increase in number and enlargement of the blood vessels in the growing bone, with solution and absorption of the osseous tissue; but that small doses diminished the normal process of absorption, leading to thickening of the bone and lessening the number and capacity of the cancelli and medullary cavities. He therefore gave it to rickety children in doses of ·0005 grams to those weighing less than 5,000 grams, and ·0001 grams to those over 10,000 grams in weight, daily for two months, and afterwards ·0005 to all for a year or more. His results were most satisfactory, not only as regards the development of the skeleton, but in relieving such nervous symptoms as laryngismus, &c.; and his experience was confirmed by Politzer, Eisenschutz, and others.

Monti and Kissel, on the other hand, have thrown doubts on the alleged physiological action of phosphorus, and they believe that it is more poisonous than is commonly supposed, and that by deranging the digestion it does actual harm.

But recently Dr. L. B. Mandelstamm has treated 450 children, carefully measuring and weighing 216, viz., 120 males and 96 females during the whole time. He continued the administration of the phosphorus for periods ranging from one or two to twelve or fifteen months. He concludes from these observations, extensive and accurate enough for all purposes, that (1) the clinical results fully confirm the favourable anticipation suggested by physiological experiments, (2) that it acts better, more certainly, and sooner than any other drug, (3) that in small doses it may be given for indefinite periods without any ill effects, and (4) that it is especially valuable in the treatment of the nervous conditions accompanying "rickets." The formula employed by both Kessowitz and Mandelstamm is

Phosphorus 0·01

Ol. Olivæ 10·0

Syrup and Mucilage 5·0

Aq. destill. 80·0

Dose, one teaspoonful three times a day, or $\frac{1}{200}$ of a grain in each dose.

